# Distributional ecology of Andes hantavirus: a macroecological approach

**DOI:** 10.1186/s12942-018-0142-z

**Published:** 2018-06-22

**Authors:** Francisca Astorga, Luis E. Escobar, Daniela Poo-Muñoz, Joaquin Escobar-Dodero, Sylvia Rojas-Hucks, Mario Alvarado-Rybak, Melanie Duclos, Daniel Romero-Alvarez, Blanca E. Molina-Burgos, Alexandra Peñafiel-Ricaurte, Frederick Toro, Francisco T. Peña-Gómez, A. Townsend Peterson

**Affiliations:** 1 Campus Huechuraba, Facultad de Ciencias, Universidad Mayor, 8580745 Santiago, Chile; 2 Department of Fish and Wildlife Conservation, Virginia Tech, Blacksburg, VA 24061 USA; 3 Centro de Investigación para la Sustentabilidad y Programa de Doctorado en Medicina de la Conservación, Facultad de Ciencias de la Vida, Universidad Andres Bello, 8320000 Santiago, Chile; 4 Escuela de Medicina Veterinaria, Facultad de Ciencias, Universidad Mayor, Santiago, Chile; 5 Department of Ecology and Evolutionary Biology, Biodiversity Institute, University of Kansas, Lawrence, KS 66045 USA; 6 Departamento de Ciencias Ecológicas, Facultad de Ciencias, Universidad de Chile, Santiago, Chile; 7 Instituto de Ecología y Biodiversidad, Facultad de Ciencias, Universidad de Chile, Santiago, Chile

**Keywords:** Andes hantavirus, Bunyaviridae, Ecological niche modeling, Maxent, Rodent reservoirs, Zoonoses

## Abstract

**Background:**

Hantavirus pulmonary syndrome (HPS) is an infection endemic in Chile and Argentina, caused by Andes hantavirus (ANDV). The rodent *Oligoryzomys longicaudatus* is suggested as the main reservoir, although several other species of Sigmodontinae are known hosts of ANDV. Here, we explore potential ANDV transmission risk to humans in southern South America, based on eco-epidemiological associations among: six rodent host species, seropositive rodents, and human HPS cases.

**Methods:**

We used ecological niche modeling and macroecological approaches to determine potential geographic distributions and assess environmental similarity among rodents and human HPS cases.

**Results:**

Highest numbers of rodent species (five) were in Chile between 35° and 41°S latitude. Background similarity tests showed niche similarity in 14 of the 56 possible comparisons: similarity between human HPS cases and the background of all species and seropositive rodents was supported (except for *Abrothrix sanborni*). Of interest among the results is the likely role of *O. longicaudatus*, *Loxodontomys micropus*, *Abrothrix olivaceus*, and *Abrothrix longipilis* in HPS transmission to humans.

**Conclusions:**

Our results support a role of rodent species’ distributions as a risk factor for human HPS at coarse scales, and suggest that the role of the main reservoir (*O. longicaudatus*) may be supported by the broader rodent host community in some areas.

**Electronic supplementary material:**

The online version of this article (10.1186/s12942-018-0142-z) contains supplementary material, which is available to authorized users.

## Background

Hantaviruses (family Bunyaviridae, genus *Orthohantavirus*) are viruses responsible for a hemorrhagic fever with renal syndrome (HFRS) worldwide, mainly in Asia and Europe, and for hantavirus pulmonary syndrome (HPS), which occurs in the Americas and is often severe or fatal in humans [1–3]. Human HPS infections occur by inhalation of aerosolized viral particles from excretions of infected rodents and, rarely, via rodent bites; infected humans develop flu-like symptoms that rapidly progress to cardiopulmonary complications, pulmonary edema, and hemodynamic failure [4–6].

In 1990, in Recife, Pernambuco (northern Brazil) HFRS cases were serologically diagnosed, representing the first cases of hantavirus disease in the Americas [7]. Later, in Baltimore, United States, 3 cases of domestically acquired HFRS were designated as caused by a local strain of Seoul virus [8]. In this region, rats (*Rattus norvegicus*) played a critical role as reservoirs of hantavirus.

In 1993, human HPS fatalities were reported in southwestern United States caused by a novel hantavirus denominated Sin Nombre virus [9]. Later, Andes virus (ANDV) was first described in 1995 after an outbreak of fatal neuropathies in Argentina [10, 11]. Chile confirmed HPS cases for the first time in the same year [10]. HPS is now recognized as an endemic disease, with obligatory notification in Argentina and Chile [10, 12, 13]. To date, ~200 cases per year associated with 25 hantavirus lineages have been reported from Canada to southern South America [1, 14, 15].

Rodents are natural hosts for hantaviruses. Typically, at least in North America, each rodent species carries a different hantavirus lineage, suggesting host-parasite co-speciation [16, 17]. In the Americas, rodent species of the subfamily *Sigmodontinae* are known to be hosts of ANDV, with *Oligoryzomys longicaudatus* as a main reservoir: this species is a common rodent in rural Chile and Argentina [10, 15, 18–20]. The shared phylogenetic history between viruses and hosts has served to predict plausible reservoirs linked to human infections [21, 22]. Estimating areas of human-risk is possible via understanding hosts species’ ecology and distribution: human cases are more likely to occur in areas overlapping with distributions of natural hosts, particularly main reservoirs [18, 23–26].

Reservoirs are defined as hosts that (1) are able to maintain an infectious agent circulating without subsidy (reinfection) from other host species, (2) tend to be tolerant to infections (i.e., do not develop serious disease), and (iii) are essential in the infectious agent’s transmission cycle [27]. A reservoir may be constituted by a single species, or by a suite of hosts that together form a reservoir [27]. Hantavirus reservoirs have been related to wild native species and synanthropic species of rodents. Indeed, the first isolation of hantavirus—a Seoul virus (SEOV)—in south American reservoirs is related to *R. norvegicus* in urban areas of Brazil and Argentina [28]. Recently, ecological niche models, which link reports of hosts or reservoirs to environmental conditions, have provided insight into hantavirus ecology and distribution (e.g., [23, 24]).

Here, we explore potential associations between rodent species’ distributions, distributions of wild native rodents of southern South America infected with ANDV, and ANDV transmission to humans. Synanthropic rodents (*Rattus* sp.) had been also associated with hantavirus transmission to humans, particularly with Seoul virus strains in the United States [7, 29], but their role in the transmission of ANDV is unclear and available data is scarce, thus, only native rodents were included in this study to reconstruct the sylvatic cycle of ANDV in southern South America. We explore eco-epidemiological associations among three actors in the ANDV transmission system: rodent host species, seropositive rodents, and human HPS cases; specifically, we used a macroecological approach to assess environmental suitability of a series of reservoirs, the virus, and the ecological similarity among the them.

## Methods

### Occurrence of rodent hosts

Six rodent species were selected as potential ANDV hosts based on reported seropositivity to hantavirus: *O. longicaudatus, Loxodontomys micropus, Phyllotis darwini, Abrothrix longipilis, A. olivaceus,* and *A. sanborni* [16, 26, 30]. Data records documenting geographic occurrences of these species were obtained from the Global Biodiversity Information Facility [31] and VertNet [32]. This information was complemented with occurrence data obtained from a detailed search of scientific literature (see below, "Occurrence of hantavirus in rodents and humans" section).

To reduce model overfit to oversampled sites and to avoid including inaccurate reports, occurrences were carefully filtered and cleaned under the following criteria: (1) remove duplicate coordinates; (2) remove incoherent reports (e.g., occurrences in the ocean or another continents); (3) mitigate oversampling by randomly sampling occurrences so that no pair was less than ~ 1 km apart [33]; (4) remove likely misidentified specimens in the form of occurrences outside species’ ranges, as defined by areas falling > 150 km from distributional areas outlined by the International Union for Conservation of Nature (http://www.iucnredlist.org) [34]; and (5) compare the country and state reported specimens with the administrative area corresponding to the geographic coordinates to detect inconsistencies. After filtering, we obtained 390 occurrence localities for *O. longicaudatus*, 189 for *L. micropus*, 74 for *P. darwini*, 137 for *A. longipilis*, 351 for *A. olivaceus*, and 20 for *A. sanborni* [31, 35–45]. Occurrence records for rodent species were reported between 1896 and 2010.

### Occurrence of hantavirus in rodents and humans

From the scientific literature, we compiled information about known occurrences of hantavirus in the rodents. Searches were conducted between July and October 2014, using scientific names of each rodent species and “hantavirus” as keywords in searches of Web of Science (www.isiknowledge.com), PubMed (www.ncbi.nlm.nih.gov), and Scientific Library Online (SciELO; www.scielo.org); for the latter, we followed the algorithm previously proposed [46]. To be included, hantavirus reports needed to include geographic location and diagnosis in laboratory facilities. Data from seropositive rodent species were merged and treated as a group, as a proxy of sites of virus exposure and circulation.

Human HPS cases were also collected from the scientific literature, searching for HPS cases on public health repositories, including the *Administración Nacional de Laboratorios e Institutos de Salud de Argentina* (ANLIS, Malbrán, C. *per comm.*), the *Instituto de Salud Pública* (ISP), and the HealthMap platform (www.healthmap.org; ProMED-mails reports [47]). We included cases reported between 1993 and 2014. We assigned geographic coordinates to sites with detailed description of the case location (e.g., municipality, town, country), or the centroid of the administrative region reported for human HPS reports for reports at municipality and locality level, allowing a maximum of 3 km of uncertainty; localities for which uncertainty was greater were excluded from analysis. For seropositive rodents, we obtained 48 reports: 34 from *O. longicaudatus*, 9 from *A. longipilis*, 3 from *A. olivaceus*, and 2 from seropositive *L. micropus;* all from Argentina and Chile between 1996 and 2006 (Additional file 1: Table S1). We obtained geographic coordinates from 311 human HPS cases [47].

### Environmental data

We used 19 climate variables from WorldClim (www.worldclim.org/) that summarize average climate conditions derived from averaged data of inland climatic stations from ~ 60 years (i.e., monthly mean, minimum, and maximum temperature and precipitation during 1950–2000), assuming that climatic patterns should be consistent with present-day conditions. These data are provided as interpolations at 30″ (~ 1 km) spatial resolution [48]. We excluded four climatic layers (bio 8, 9, 18, and 19), since these variables include artifacts that create abrupt differences between neighboring pixels [48]. We carried out a principal components analysis from the WorldClim variables for each model; i.e., models of each rodent species, seropositive rodents, and human cases, retaining enough components to summarize ≥ 99.9% of total environmental variance [33]. The first three components were also used to generate an environmental space in which to visualize occurrence records using NicheA v. 3.0 [49, 50].

### Ecological niche modeling

The analysis extent was set individually for each species as a hypothesis of each species’ accessible area M (sensu [51]); this choice has important effects on ecological niche modeling outputs [52]. We estimated specific areas of analysis for each rodent species, seropositive rodents, and human cases, for a total of eight model experiments. We defined the analysis area as a 220 km buffer around each occurrence set, dissolving the resulting polygons to outline a continuous area [53].

We created 10 replicate models for each species by randomly subsampling 70% of occurrences, to account for sampling effects in the occurrence data [54]. For each model replicate, we split occurrences randomly into two subsets: one for model calibration (75% of occurrences), and another for model evaluation (25%). These steps allowed us to assess model uncertainty quantitatively.

Ecological niche models were calibrated in Maxent version 3.3.3 k [55]. Specific settings included 10 bootstrap replicates, random seed, and median of the 10 replicates in logistic format as output. The logistic output was interpreted here conservatively as a suitability index rather than as a probability [56]. To evaluate model predictions, continuous outputs were converted to binary maps based on the highest suitability threshold that included 95% of the calibration occurrences (i.e., E = 5%; [33]); this threshold considers the amount of error (E) likely in the occurrence data. As an evaluation metric we applied a cumulative binomial probability test (α = 0.05) to the binary maps [33]: number of evaluation occurrences was used as number of trials, number of evaluation occurrences predicted correctly were used as the number of successes, and the proportion of the evaluation area predicted suitable was used as the null probability of a success [57]. Replicate models with the lowest p-values were selected as final models, and used in succeeding analyses.

Finally, because host species richness may be an important element in the ecology of infectious diseases [58], we developed a hantavirus host-species richness map. Specifically, we assembled the final ecological niche model of each rodent species. Model assemble was done by summing the binary maps of the rodents’ potential distribution.

### Background similarity test

We assessed whether ecological niche models from each of the six rodent species, human HPS cases, and seropositive rodents were statistically distinguishable or not at the spatial resolution of our analyses [59]. Ecological niche similarity was measured using ENMtools software version 1.4.3, based on the Schoener’s D index [60]. Index values from observed models were compared against null distributions (see below) to assign probability values to observed values of similarity [59]. Null distributions were developed in ENMTools using Maxent to test whether each pair of ecological niche models was statistically undistinguishable (not different), considering the background (= M) for each model. The background similarity test [60] compares the observed similarity of a species pair to the similarity between one of the species and random points from the background (M area) of the other species. This process was repeated 100 times, comparing each species against the background of the other in each species pair [60].

## Results

### Ecological niche modeling

**Fig. 1:**
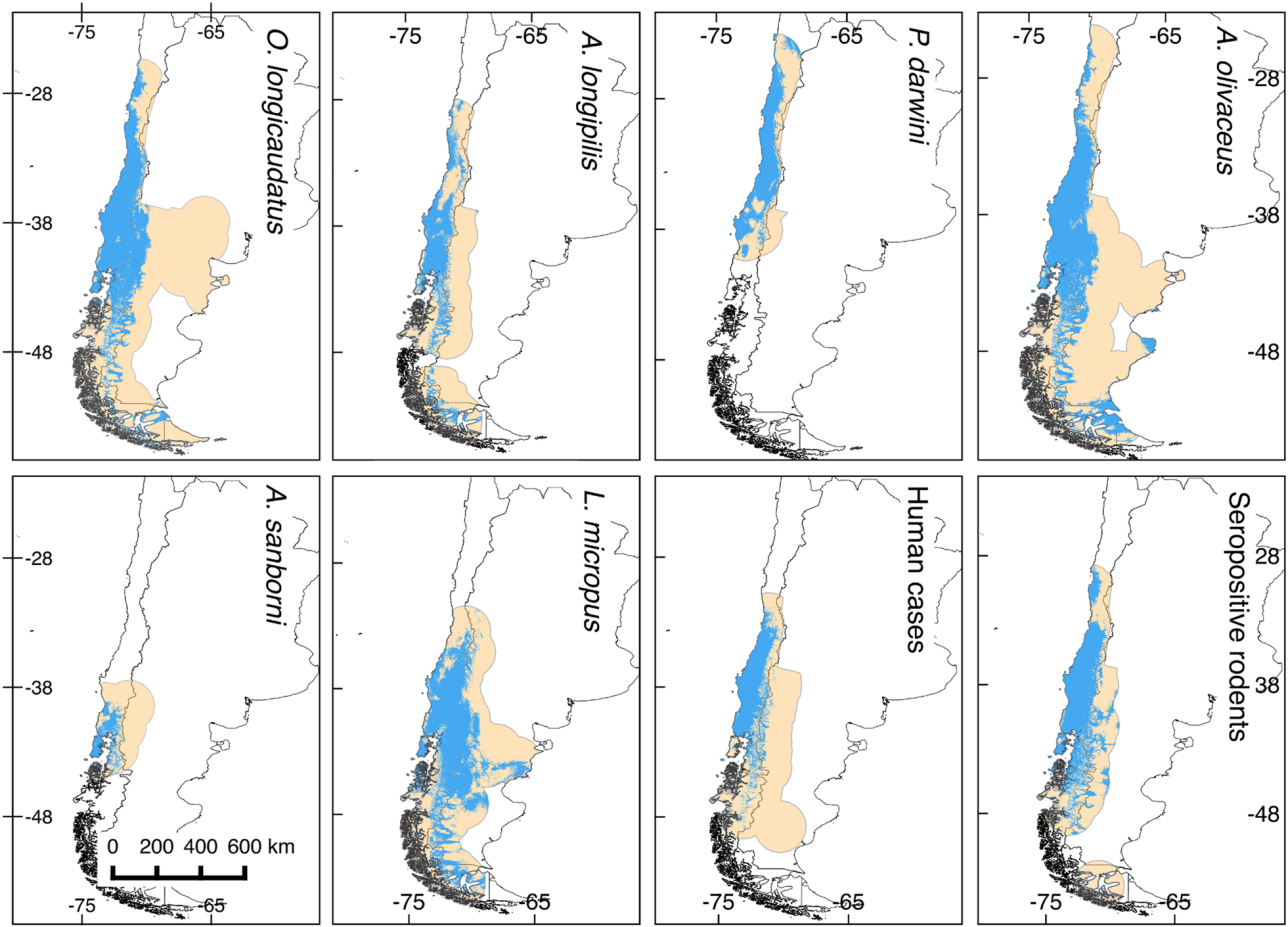
Ecological niche models for rodent hantavirus hosts, seropositive rodents, and human HPS cases. Orange areas depict potential distributions based on ecological niche models. Blue areas show the study area M for model calibration. These binary maps were generated based on an acceptable omission rate of 5%

**Fig. 2:**
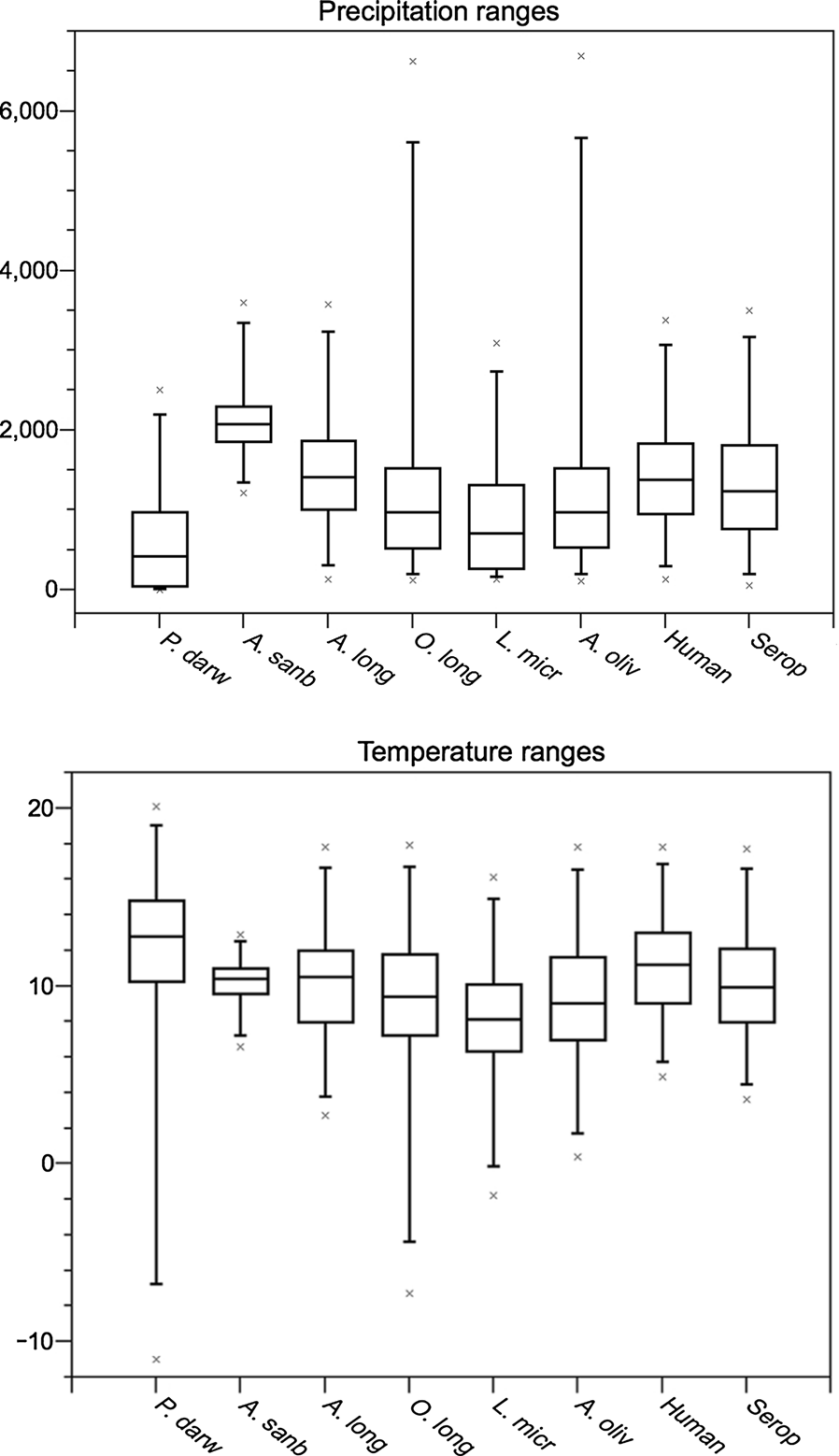
Temperature and precipitation tolerances derived from niche models for six species of rodent hosts, infected rodents, and human HPS cases, based on ecological niche models. Boxplot figures depict precipitation (in mm) and temperature (in  °C degrees) intervals occupied by each species or group analyzed

**Fig. 3:**
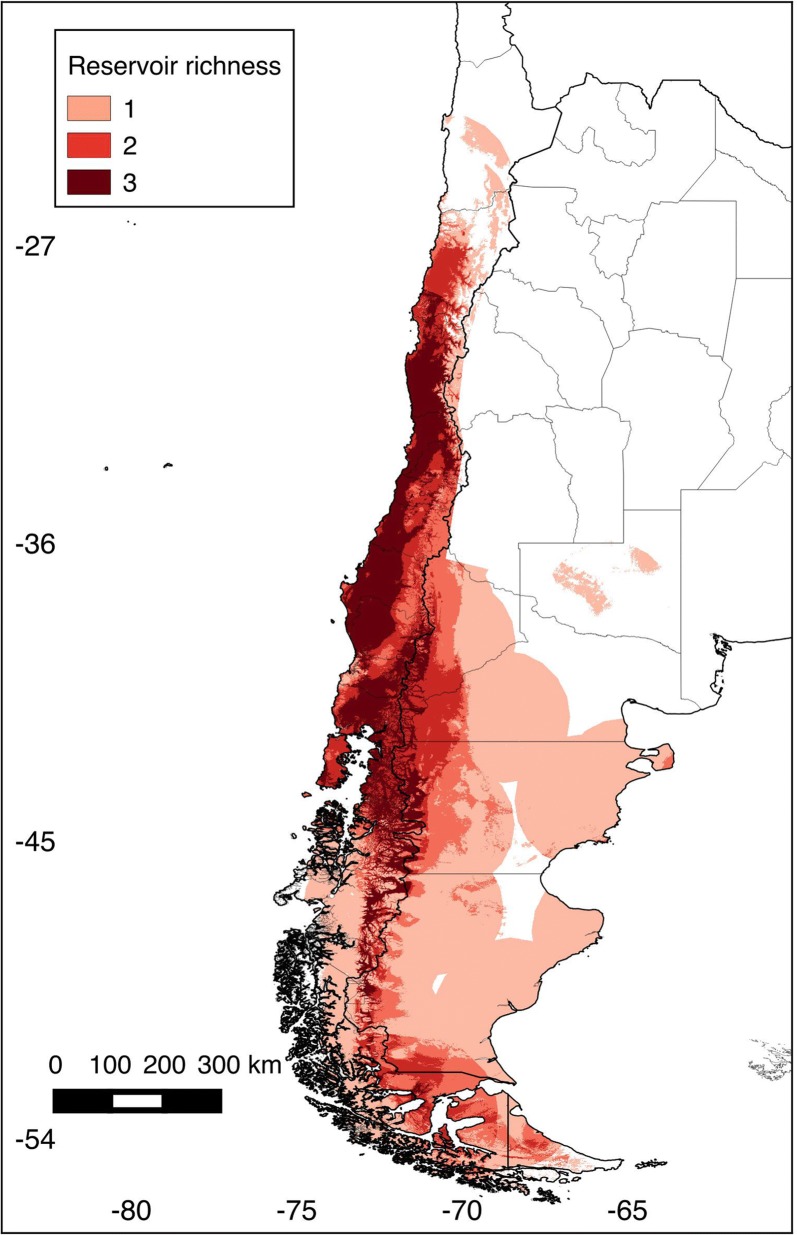
Rodent species richness (number of species predicted by cell). Chile and Argentina, divided by Regions (Chile) and Provinces (Argentina). Areas of high (dark red) to low (light pink) richness of rodent hosts were identified according to ecological niche model predictions. Values represent the number of rodent species by pixel as predicted by the ecological niche models

Eight niche models were generated in this study: six for rodents, one for seropositive rodents, and one for human HPS cases (see map in Fig. 1). All models predictions were statistically better than random expectations (p < 0.05). Among rodent models, median annual temperature ranged from 7.9 °C (*L. micropus*) to 13.1 °C (*P. darwini*) (Fig. 2); median precipitation among rodent models ranged from 440 mm (*P. darwini*) to 2127 mm (*A. sanborni*). *Phyllotis darwini* model showed potential distribution in areas with lower precipitation (16–1599 mm, Fig. 2) than other species. In general, *O. longicaudatus* and *A. longipilis* showed broader ranges of tolerance to precipitation and temperature than other species. Highest rodent species richness was in Chile between 35° and 41°S latitude (Fig. 3), with suitable conditions and accessible sites for five species.

### Ecological niche model comparisons

**Fig. 4:**
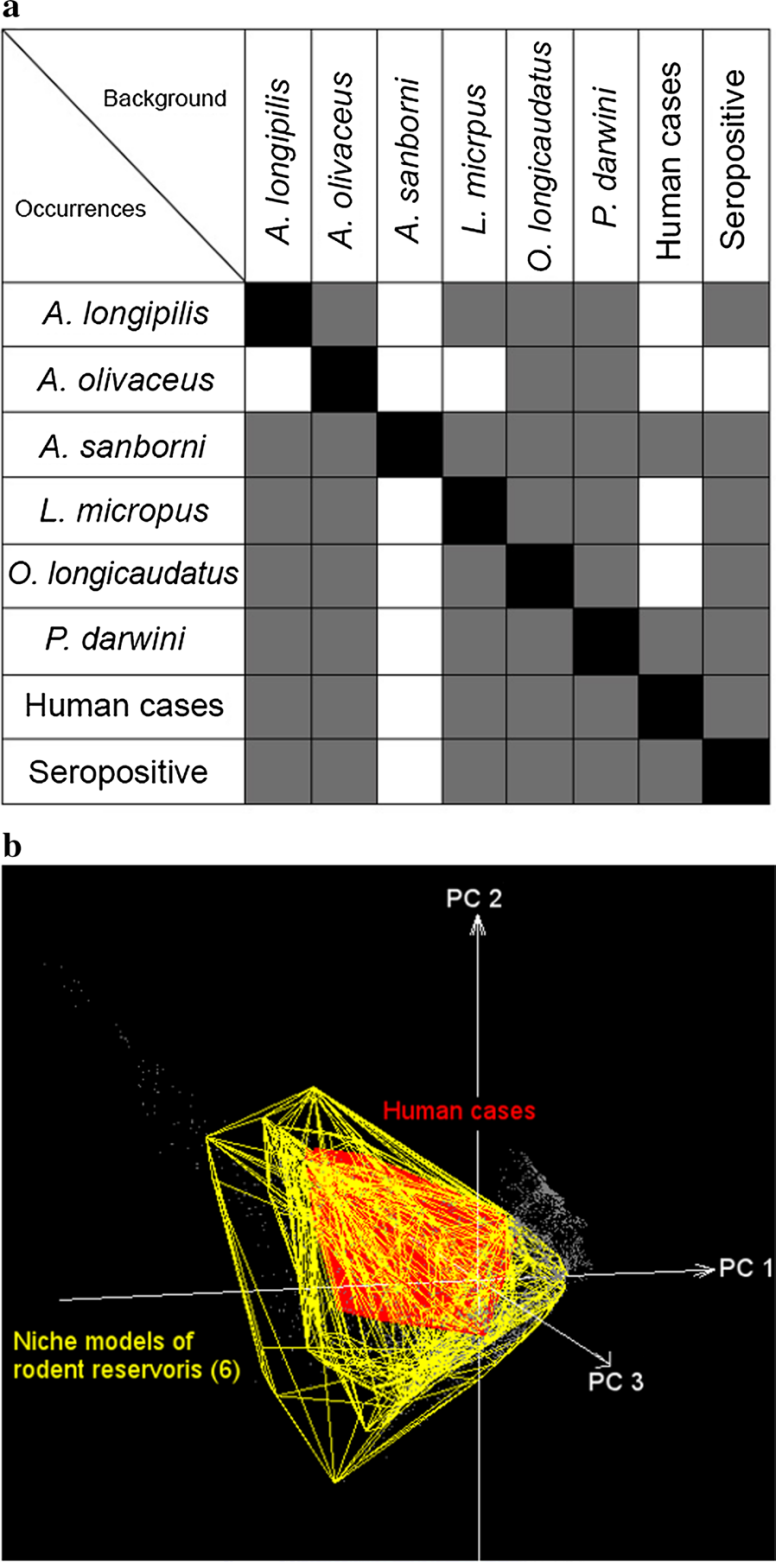
Ecological niche similarity tests:** a** Background similarity tests were developed in a series of two-way comparisons. Occurrences (*y*-axis) were compared against the backgrounds of each other species (*x*-axis). Gray fill indicates that the null hypothesis of no difference between niches was not rejected (*p* > 0.05), and white squares denote hypothesis rejected;** b** Convex polyhedrons derived from occurrences of each rodent species (yellow) and human HPS cases (red) in a multidimensional environmental space (principal components 1, 2, and 3, obtained from the original bioclimatic layers). Note that the environmental space occupied by human cases are contained within the set of environments used by the rodent species

**Fig. 5:**
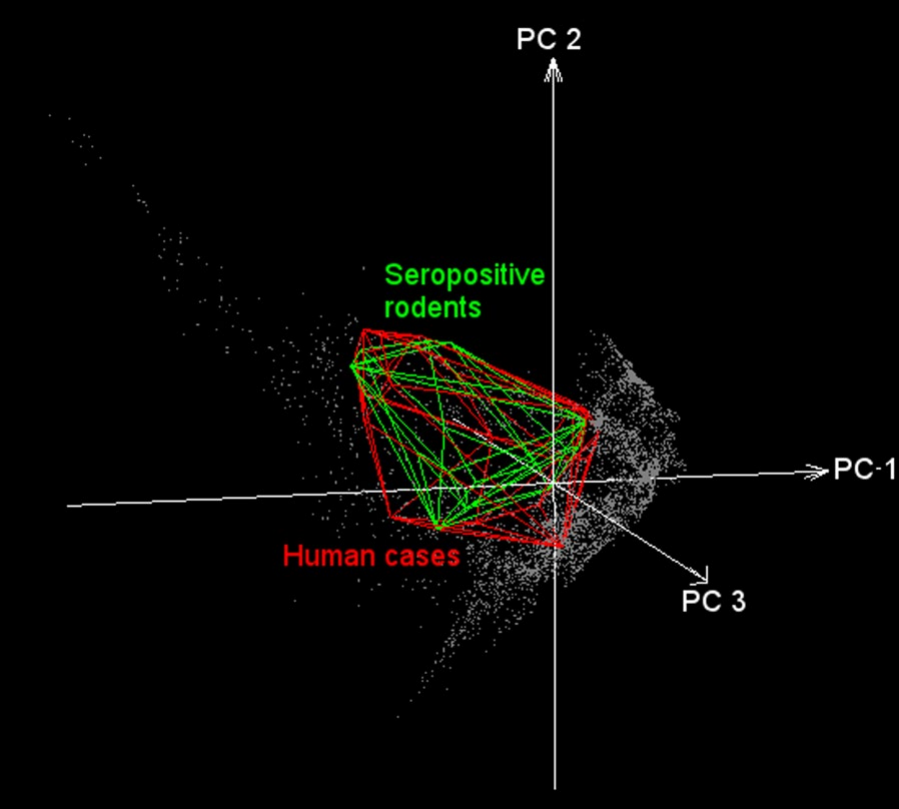
Hantavirus ecological niche model visualized in environmental space. Model predictions of human HPS cases (red) and seropositive rodents (green) displayed in a multidimensional environmental space (principal components 1, 2, and 3 obtained from the original bioclimatic layers). Note the considerable overlap of environments occupied by human cases and hantavirus seropositive rodents. This suggests that the presence of seropositive rodents may explain and predict spillover events (transmission of hantavirus from rodents to humans)

Based on background similarity tests we accepted the null hypothesis of no difference between niche models in 14 of the 56 possible comparisons (Fig. 4a). In all comparisons against the *A. sanborni* background, the null hypothesis of no difference was rejected. Background similarity tests also failed to detect any difference between human HPS and the background of any rodent species (Fig. 4b), except for *A. sanborni* (Fig. 4a), or the background of seropositive rodents (Fig. 5). The no difference hypothesis was rejected for comparisons of seropositive rodents against the background of *A. olivaceus* and *A. sanborni*.

## Discussion

Hantavirus-induced HPS has been recognized as a significant zoonotic disease and threat to public health across the Americas [1]. In spite of important improvements in diagnosis and surveillance methods in South America, however, hantavirus transmission dynamics remain incompletely characterized [61]. This study, which compiles considerable information relevant to ANDV distribution in Chile and Argentina, aims to lay a foundation for a deeper understanding of this disease in southern South America.

Of particular interest among our results were the roles of *O. longicaudatus*, *L. micropus*, *A. olivaceus*, and *A. longipilis*, in risk of transmission to humans, given associations between these rodent species and human cases and rodent seropositivity. To document hantavirus infections, we used rodent seroprevalence (i.e., rodent exposure to the virus) and human HPS cases, both valid and complementary indicators of hantavirus transmission. However, geolocation of human infection sites may be much less accurate, as symptoms take days or weeks to manifest [1]. Thus, human HPS case data may at times provide incorrect or overly general signals on the ecology of hantavirus. Human HPS cases represent an integration of all elements of the transmission system, forming -in effect a black box [33].

By including all components of the transmission cycle in this study hosts, virus-exposed hosts, and terminal host infections (i.e., in humans), we explored different components of the distributional ecology of hantavirus; specifically, we quantified the potential distribution of six recognized native rodent reservoirs of the virus in southern South America. This is a macroecological study since it assessed biodiversity patterns at coarse spatial scale, which provides new information regarding suitable conditions for hantavirus transmission risk and rodent species likely involved in local transmission. In general, we argue that host distributions influence pathogen distributions, thereby molding the occurrence of the disease [18], which may be explained by the subset of the rodent niche occupied by hantavirus (Fig. 4b). This assessment may be useful for native (non-introduced) infectious disease such as ANDV hantavirus, since introduced diseases in complex multihost systems such as plague may have geographic occurrence determined by the environmental conditions of the pathogen per se and not necessarily by the range of the hosts [62].

**Fig. 6:**
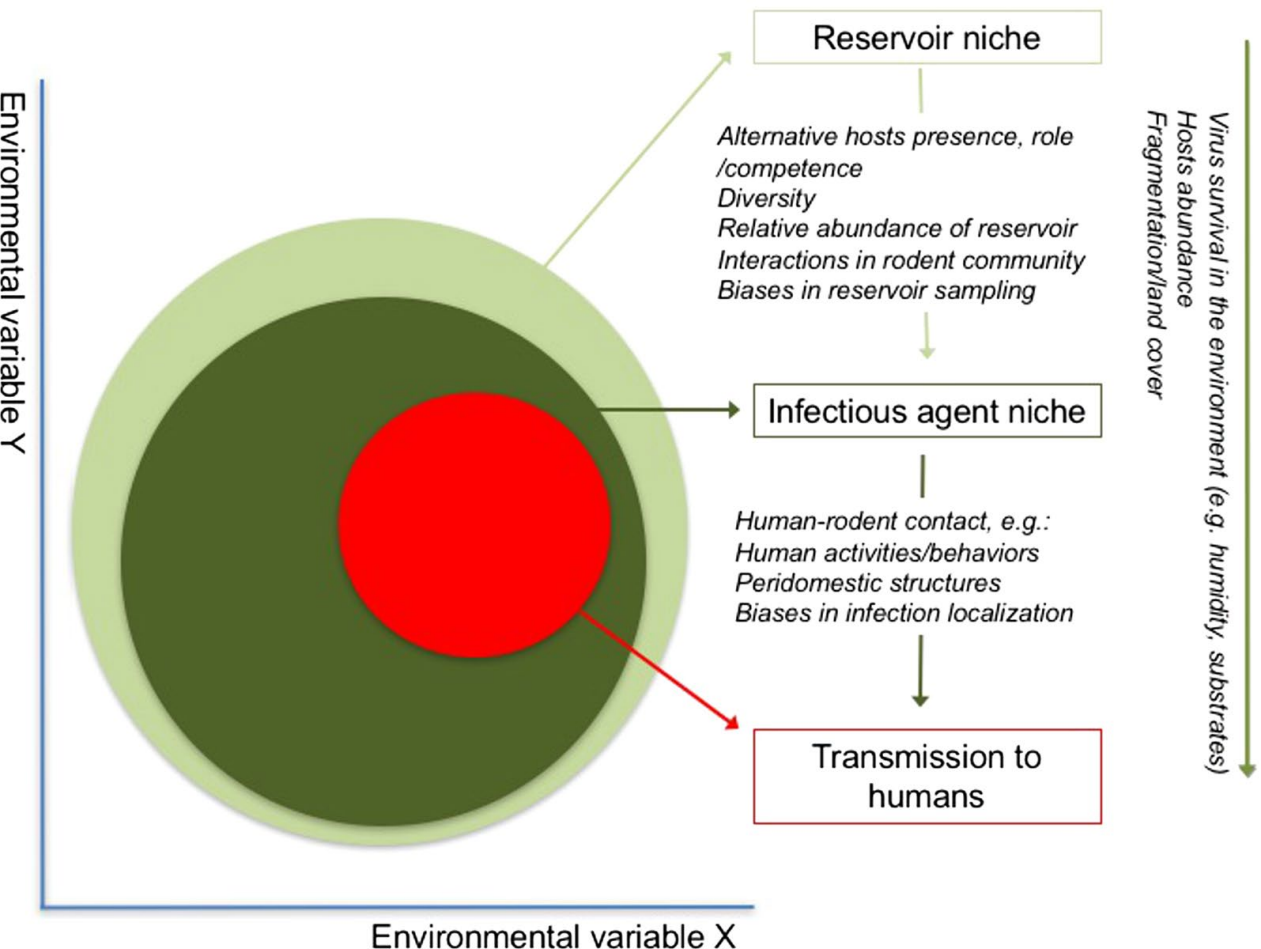
Proposed framework for the hantavirus system. The figure illustrates three scales (rectangles) of hantavirus occurrence. The reservoir’s niche comprises the set of abiotic environmental conditions required by each of the rodent species to maintain populations in the long term (light green circle). Hantavirus’ niche is the set of suitable abiotic environmental conditions necessary for hantavirus at coarse (e.g., climate) and fine scales (e.g., host internal temperature; dark green circle). Finally, a “niche” may characterize conditions apt for transmission to humans: sites where rodents and hantavirus co-occur, and in which susceptible humans are able to be infected (red circle). Elements influencing gaps between scales are shown in italics. At the right, elements that may affect overall processes

In the case of hantavirus, rodent hosts are exposed unevenly to the virus across their geographic distributions [63], but in environmental terms, seropositive rodents and humans overlap considerably (Fig. 5). Consequently, seropositive rodent may represent the manifestation of hantavirus circulation in the ecosystem and human HPS cases would be manifestation of past spillover events, thus, their niche similarity suggests that spillover occurs under specific tractable and consistent environmental conditions [64, 65] (Fig. 5). Considering this framework, three ecological levels are involved: (1) reservoir niche, (2) infectious agent niche, and (3) spillover event (transmission to humans), where some variables may influence all levels (e.g. humidity), whereas others may affect only certain levels (e.g. spillover influenced by human-rodent contact; Fig. 6). Below, we discuss potential interpretations and limitations of the patterns detected in our models.

### Human HPS cases and rodent seroprevalence

Seropositive rodents and human HPS cases were indistinguishable in terms of their environmental signatures (Fig. 5), although the seropositive rodents occupied broader geographic areas and environmental space (Fig. 1: Seropositive rodents and human cases, and Fig. 5). Thus, as described elsewhere [63], human cases are not determined only by the presence of infected rodents [63, 64]. Rather, other factors may increase the exposure to the virus at local scale [64]. In northern Chile and Argentina, dry environmental conditions, may directly affect hantavirus viability in the local environment [66]. In contrast, in southern Chile and Argentina, mixed evergreen-deciduous temperate forests and humid conditions may facilitate virus survival, facilitating indirect transmission [61, 67].

Other fine-scale factors that influence human transmission are beyond the scope of the present study, but may be crucial for transmission. Such factors include rodent abundance [68], human behavior (e.g., farming, tourism), human and rodent immunity, human social status [63, 69], and the quality of human housing and peridomestic structures, among others [39, 63, 64, 70–75]. In some areas where infected rodents are distributed, humans may not be present, or may be present in lower densities [64]; such could be the case in northern Argentina and Chile, in areas occupied by important rodent reservoirs such as *O. longicaudatus* (Fig. 1), but with low human densities [76]. These areas are thus of low public health concern in terms of few HPS cases, although risk for the few humans present may be significant. We note that all these patterns may be further complicated by inaccurate diagnoses and incomplete reporting, which are significant problems for hantavirus detections in humans [77].

Our exploration was temporally static, yet hantavirus transmission may have a significant seasonal dimension. For example, most human HPS cases in Chile and Argentina are reported during spring and summer, when rodent abundances and seroprevalences tend to be high [62, 64, 75, 78, 79]. Additionally, many human activities concentrating human presence in situations of risk, such as farming and tourism, may occur chiefly in summer [26, 39, 64]. On coarser time scales, climatic events such as the El Niño Southern Oscillation affect population dynamics of rodents and have been tied to human HPS outbreaks [80–84]. Our coarse scale ecological niche models offer novel information regarding climatic factors associated with host rodent distributions and hantavirus incidence over broad regions, pointing to high-priority areas where further studies can be developed to include fine-resolution temporal and spatial variables.

### Human HPS cases and rodent distributions

In terrestrial (Fig. 1) and environmental spaces (Fig. 4b), the model of human HPS was nested within the modeled distributions and niches of all rodent species. In other words, our results support a role for these rodent species as risk factors in human HPS, and that HPS cases appear to be restricted by the geographic distribution of the rodent hosts (Fig. 5). Previous studies have proposed that the geographic distribution of the main reservoir constrains the distribution of hantavirus [5, 18, 38, 68]; however, our results do not support this “host-niche hypothesis” completely [58]. That is, we were unable to identify a single main reservoir that explains the distribution of the virus across its estimated range. Rather, several rodent species had niche models and potential geographic distributions overlapping those of seropositive rodents and human HPS cases (Figs. 1, 4, 5). As such, other species may be associated with transmission to humans, or with persistence of the virus at sites where human infection occurs. Alternatively, we found patterns more consistent with a “pathogen niche hypothesis” [58], in which hantavirus occupies a specific environmental space; thus, ideal rodent hosts are those with the highest overlap, spatially or environmentally with the hantavirus [58]. To test this hypothesis, further studies should focus on sampling areas where *O. longicaudatus* and other potential reservoir candidates are present, but no hantavirus detections exist. This information will allow understanding whether the absence of hantavirus detection is caused by environmental conditions or limited surveillance effort. Finally, even though the focus of the study was ANDV and HPS cases in terms of native reservoirs, it is important to highlight that other hantavirus lineages can cause HPS and that other rodent species may play a role in the transmission of hantavirus to humans in southern South America. Thus, the exploration of other hantavirus lineages circulating in other rodent species, specially synanthropic rats [85], is warranted.

## Conclusions

Our results support a role of rodent species’ distributions as a risk factor for human HPS at coarse scales, and suggest that the role of the main reservoir (*O. longicaudatus*) may be supported by the broader rodent community.

## Additional file

**Additional file 1.** References and sources used to collect geographic coordinates of rodent hosts, seropositive rodents and human HPS cases.
