# Supplementary material for: Distributional ecology of Andes hantavirus: a macroecological approach
Source: Int J Health Geogr. 2018 Jun 22;17:22. doi: 10.1186/s12942-018-0142-z (PMC6013855; doi:10.1186/s12942-018-0142-z)
Supplement: Supplementary file 1 — Additional file 1. References and sources used to collect geographic coordinates of rodent hosts, seropositive rodents and human HPS cases. [file 12942_2018_142_MOESM1_ESM.docx]

# Table S1. References and sources used to collect geographic coordinates of rodent hosts, seropositive rodents and human HPS cases.

| **Model** | **Source** |
| --- | --- |
| All rodents | [1] |
| *L. micropus* | [2, 3] |
| *A. olivaceus,* seropositive rodents | [4] |
| *A. olivaceus* | [5] |
| *A. sanborni*, seropositive rodents, *O. longicaudatus* | [6] |
| Seropositive rodents, *O. longicaudatus* | [7, 8] |
| Seropositive rodents | [9–12] |
| *O. longicaudatus* | [13–17] |
| *P. darwini* | [18] |
| Human HPS cases | [19] |

**References**

1. GBIF. Global Biodiversity Information Facility. Available online: http://www.gbif.org. Accessed 17 July 2014.
2. Sauthier D, Teta P, Wallace P, Pardiñas UFJ. Mammalia, Rodentia, Sigmodontinae, *Loxodontomys micropus*: New locality records. Check List, 2008; doi:10.15560/4.2.171.
3. Cañón C, D’Elía G, Pardiñas UFJ, Lessa EP. Phylogeography of *Loxodontomys micropus* with comments on the alpha taxonomy of *Loxodontomys* (Cricetidae: Sigmodontinae). J. Mammal, 2010; doi:10.1644/10-MAMM-A-027.1.
4. Ortiz JC, Venegas W. Estudio e identificación de las especies de roedores silvestres reservorios del virus Hanta en la Región del Bío-Bío. Informe Final; Concepción, Chile, 1998.
5. Monjeau JA, Rotela CH, Lamfri M, Márquez J, Scavuzzo CM, Stanulescu M, Nabte MJ, Rial EG. Estimating habitat suitability for potential hantavirus reservoirs in north-western Patagonia using satellite imagery: Searching for the best predictive tools. Mamm. Biol. 2011; doi:10.1016/j.mambio.2011.04.001.
6. Torres-Pérez F, Navarrete-Droguett J, Aldunate R, Yates TL, Mertz GJ, Vial PA, Ferrés M, Marquet PA. Peridomestic small mammals associated with confirmed cases of human hantavirus disease in southcentral Chile. Am J Trop Med Hyg. 2004;70:305-9.
7. Belmar-Lucero S, Godoy P, Ferrés M, Vial P, Palma RE. Range expansion of *Oligoryzomys longicaudatus* (Rodentia, Sigmodontinae) in Patagonian Chile, and first record of hantavirus in the region. Rev Chil Hist Nat*.* 2009; doi:10.4067/S0716-078X2009000200008.
8. Larrieu E, Cantoni G, Herrero E, Pérez A, Talmon G, Vázquez G, Arellano O, Padula P. Hantavirus antibodies in rodents and human cases with pulmonary syndrome, Rio Negro, Argentina. Med. 2008; 68:373-9.
9. Padula P, Figueroa R, Navarrete M, Pizarro E, Cádiz R, Bellomo C, Jofré C, Zaror L, Rodríguez E, Murúa R. Transmission study of Andes Hantavirus infection in wild Sigmodontine rodents. J Virol. 2004; doi:10.1128/JVI.78.21.11972.
10. Torres-Pérez F, Palma RE, Hjelle B, Holmes EC, Cook JA. Spatial but not temporal co-divergence of a virus and its mammalian host. Mol Ecol. 2011; doi:10.1111/j.1365-294X.2011.05241.x.
11. Cantoni G, Padula P, Calderón G, Mills JN, Herrero E, Sandoval P, Martínez V, Pini N, Larrieu E. Seasonal variation in prevalence of antibody to hantaviruses in rodents from southern Argentina. Trop. Med. Int. Heal. 2001; doi:10.1046/j.1365-3156.2001.00788.x.
12. Servicio Agrícola y Ganadero. Aplicación monitoreo y determinación reservorios múridos de hantavirus en la Región de Aysén. SAG-FNDR 1999-2002. Santiago, Chile; 2002.
13. Palma RE, Rivera-Milla E, Salazar-Bravo J, Torres-Pérez F, Pardiñas UFJ, Marquet P, Stoporno AE, Meynard A, Yates T. Phylogeography of *Oligoryzomys longicaudatus* (Rodentia: Sigmodontinae) in temperate South America. J Mammal. 2005; doi:10.1644/1545-1542(2005)086<0191:POOLRS>2.0.CO;2.
14. Teta P, Pereira JA, Fracassi NG, Bisceglia SBC, Fortabat SH. Micromamíferos (Didelphimorphia y Rodentia) del Parque Nacional Lihué Calel, La Pampa, Argentina. Mastozoología Neotrop*.* 2009;16:183–98.
15. Pardiñas UFJ, Teta P. Micromamíferos del sector oriental de la altiplanicie del Somuncurá (Río Negro, Argentina). Mastozoología Neotrop. 2007; 14:271–8.
16. González LA, Murúa R, Jofré C. Habitat utilization of two muroid species in relation to population outbreaks in southern temperate forest of Chile. Rev Chil Hist Nat. 2000; doi:10.4067/S0716-078X2000000300012.
17. Ortiz JC, Venegas W, Sandoval JA, Chandia P, Torres-Pérez F. Hantavirus in rodents of the VIII Region of Chile. Rev Chil Hist Nat. 2004; doi:10.4067/S0716-078X2004000200005.
18. Crespín L, Lima M. Supervivencia adulta y dinámica poblacional del lauchón orejudo *Phyllotis darwini* en Chile central. Rev. Chil. Hist. Nat. 2006; doi:10.4067/S0716-078X2006000300002
19. Pro-MED Program for Monitoring Emerging Diseases- mail. Available online: http://www.promedmail.org. Accessed 05 Sept 2014.
